# Progress of Obstetrics

**Published:** 1875-07

**Authors:** E. Warren Sawyer

**Affiliations:** Lecturer on Obstetrics, Rush Medical College, Chicago


### Progress in Medical Sciences.

ARTICLE I.

### PROGRESS OF OBSTETRICS.

BY E. WARREN SAWYER, M.D.,

LECTURER ON OBSTETRICS, RUSH MEDICAL COLLEGE, CHICAGO.

### MANUAL DILATATION OF THE OS UTERI.

In the Boston Medical and Surgical Journal, Feb. 4, Dr. Sinclair has called attention to this mode of dilating the mouth of the uterus in the report of a series of cases in which the hand only was used. Nine cases in all are mentioned: "One of convulsions at the seventh month, three of convulsions at the eighth month, two of placenta prævia at the eighth month, one of uterine hæmorrhage at the fifth month, one of accidental hæmorrhage in the ninth month, and one of persistent vomiting at the seventh month." In all of which it was decided to induce premature expulsion of the fœtus.

The writer says, that "wherever a sponge tent or rubber bag can be inserted, the finger can be introduced." When a single finger has opened the cervix all it is capable of, a second may be added to this, and then a third, and fourth and thumb, and then the hand is shaped into a wedge which may be gently insinuated through the cervix into the cavity of the body. In the cases reported, from fifteen minutes to an hour and a half was required to complete the dilatation.

In the *Traite complet de l'art des accouchemens*, Paris, 1833, Velpeau has spoken of dilatation of the cervix with the fingers, and says that instrumental dilatation is unnecessary ; further, if the neck is the seat of disease, as of scirrhus, it is useless to attempt this method.

In three of Dr. Sinclair's cases the patient was anæsthetized during the operation ; in *all* instances it seems that ether would facilitate the dilatation—at any rate, ether should be given to relieve the pain incident to the introduction of the hand into the vagina, which is frequently an agonizing operation.

### INTRA-UTERINE AMPUTATION.

At a recent meeting of the Dublin Obstetrical Society, Dr. Macan gave another instance of this unusual occurrence. In this case, the boy was born at term and weighed 6 lbs., 12 oz. The amputation had taken place through the left forearm, just below the insertion of the biceps. The amputated limb was not found. There was a cicatrix on the stump, which had evidently been a long time healed ; just beneath this, but not adherent, the bone was felt. The mother was a healthy young woman who had given birth to five well formed children previously; nothing unusual was observed during this pregnancy.

### CONGENITAL DEFORMITIES.

In a recent lecture at Harvard, (Boston Med. and Surg. Journal, April 22,) Dr. Buckingham said : "Concerning what are usually called mother-marks, the result of the so-called longings, or of frights during pregnancy, I assure you that I never yet saw one when I had asked, previous to the delivery, if anything of the kind was anticipated. For years it was my habit to ask the question before labor began, 'if any marks were expected.' Never but in one instance did I have an answer in the affirmative which was followed by any appearance like that which was expected. The case was this : Mrs. —— said to me, that when about six months pregnant, while she was at the theatre, a man upon the stage was apparently shot just above the nose, and the blood flowed from the spot. She knew that her child was marked by it. To my astonishment, when the baby was born, there was a red spot covering the lower part of the forehead, between the eyes and down to the nose. Of course, I had no explanation to make. On the following day, however, the patient's mother relieved me very much when she came into the room, uncovered the little one to look at it, and exclaimed, 'Why, Eliza, she's got just such a mark as you had when you were born.' It was an inherited spot, and, like very many of these marks, faded away as the child grew older."

---

NOTE.

Much comment has been recently elicited on account of a fatal case in England, of rupture of the vagina, which, from a professional standpoint, was, to say the least, remarkable. The facts are briefly these : A Mr. Peacock attended a woman in confinement ; after the labor was over, he found a coil of intestines prolapsed through the vagina which he cut off, placed in a chamber and afterwards in a privy. The woman died two hours after the operation. The coroner directed an examination of the body to be made, when it was discovered that a rent was made through the vagina, large enough to admit the hand, and that fifteen feet of the large and small intestines and mesentery were wanting. At the inquest, Dr. Barnes testified favorably to the defendant. The jury concluded that there was no evidence of "gross ignorance, want of skill, or gross inattention," and returned the verdict : "That the deceased died from the effect of an escape of intestines, consequent upon spontaneous rupture of the vagina during her confinement." Despite this, however, on behalf of the husband, Mr.

Peacock was indicted for manslaughter, tried and convicted, and is now serving out a term of six months imprisonment to which the court sentenced him. The Obstetrical Journal says of this unfortunate case, "From the excellent character, both moral and professional, which Mr. Peacock received, there appeared to be no grounds for believing that the laceration which ended so disastrously was caused by any want of care or skill."

